# The Empathy and Systemizing Quotient: The Psychometric Properties of the Dutch Version and a Review of the Cross-Cultural Stability

**DOI:** 10.1007/s10803-015-2448-z

**Published:** 2015-04-25

**Authors:** Y. Groen, A. B. M. Fuermaier, A. E. Den Heijer, O. Tucha, M. Althaus

**Affiliations:** Clinical and Developmental Neuropsychology, Faculty of Behavioral and Social Sciences, University of Groningen, Grote Kruisstraat 2/1, 9712 TS Groningen, The Netherlands; Child and Adolescent Psychiatry, University Medical Center Groningen, University of Groningen, Hanzeplein 1, 9713 GZ Groningen, The Netherlands

**Keywords:** EQ, SQ, Extreme male brain hypothesis, Empathy, Theory of mind, Sex differences

## Abstract

The ‘Empathy Quotient’ (EQ) and ‘Systemizing Quotient’ (SQ) are used worldwide to measure people’s empathizing and systemizing cognitive styles. This study investigates the psychometric properties of the Dutch EQ and SQ in healthy participants (n = 685), and high functioning males with autism spectrum disorder (n = 42). Factor analysis provided support for three subscales of the abridged 28-item EQ: Cognitive Empathy, Emotional Empathy and Social Skills. Overall, the Dutch EQ and SQ appeared reliable and valid tools to assess empathizing and systemizing cognitive style in healthy adults and high functioning adults with autism. The literature showed good cross-cultural stability of the SQ and EQ in Western countries, but in Asian countries EQ is less stable and less sensitive to sex differences.

## Introduction

According to the empathizing–systemizing theory (E–S theory) of sex differences (Baron-Cohen 2009), *empathizing* is defined as “the drive to identify another person’s emotions and thoughts, and to respond to these with an appropriate emotion” (Baron-Cohen 2002). According to the theory, the complementary cognitive style of empathizing is *systemizing,* which is the drive to (1) analyse the variables in a system, (2) to derive the underlying rules that govern the behaviour of a system, and (3) to construct systems (Baron-Cohen 2002). Systemizing allows a person to predict and control the behaviour of a system. Approximately one decade ago, two self-report questionnaires were introduced to measure the extent to which people possess these cognitive styles; the Empathy Quotient (EQ) (Baron-Cohen and Wheelwright 2004) and the Systemizing Quotient (SQ) (Baron-Cohen et al. 2003). To date, numerous studies have found that females adopt on average a more empathizing style, while males adopt on average a more systemizing style of information processing, with sex differences reaching effect sizes of half to one standard deviation. The E–S theory distinguishes different brain types that can be determined by means of the standardized scores on the EQ and SQ (Baron-Cohen 2002; Wheelwright et al. 2006). Individuals with higher standardized scores on the EQ than the SQ are categorized as having an empathizing or ‘female brain’ (type E), whereas individuals with higher standardized scores on the SQ than the EQ are categorized as having a systemizing or ‘male brain’ (type S). Individuals having equal standardized scores of the EQ and SQ are categorized as having a ‘balanced brain’ (type B). Consequently, the difference score (D) of the EQ and SQ can be used to characterize a person’s cognitive style or brain type.

The E–S theory originated from the research on autism spectrum disorder (ASD) (Baron-Cohen et al. 1985; Baron-Cohen 2009). Individuals with ASD are characterized by difficulties in social interaction and communication, alongside with unusually strong and narrow interests and repetitive behaviour (American Psychiatric Association 2013). Early theories explained the social and communicative difficulties of individuals with ASD by “mind-blindness”, which is the inability to put oneself into someone else’s shoes, to imagine their thoughts and feelings (Baron-Cohen et al. 1985). The E–S theory extended this mind-blindness theory by adding difficulties in emotional reactivity, forming the empathizing factor, and by adding the systemizing factor that could also explain the non-social characteristics of the disorder (such as the narrow interests and attention to detail) (Baron-Cohen 2002, 2009). According to this theory, individuals with ASD lie at the extreme end of the normally distributed difference between systemizing and empathizing (D), and consequently possess an above average systemizing cognitive style but a low and/or deficient empathizing style, i.e. an extreme type S or extreme male brain. A large number of studies making use of the EQ and/or SQ provided support for this Extreme Male Brain (EMB) hypothesis in ASD by demonstrating that males report lower levels of EQ, higher levels of SQ and hence a more systemizing brain type than females, while patients with ASD (both males and females) report even lower levels of EQ, even higher levels of SQ and even more systemizing brain type than males (Baron-Cohen and Wheelwright 2004; Baron Cohen et al. 2014; Berthoz et al. 2008; Sucksmith et al. 2013; Wakabayashi et al. 2007; Wheelwright et al. 2006). Moreover, autistic traits as measured by the Autism Spectrum Quotient (AQ) could be successfully predicted by both EQ and SQ in a community sample as well as in a sample of patients with ASD (Wheelwright et al. 2006). For both groups factor analysis had demonstrated that EQ and SQ both had strong loadings on AQ and together accounted for ~75 % of variance in the AQ scores. The EMB-hypothesis is further supported by the results of neuropsychological studies performed on children with ASD, demonstrating poorer performance on tests of social cognition (e.g. the ‘seeing leads to knowing test’, the ‘false belief test’ and the ‘reading the mind in the eyes test’) compared to typically developing children, and intact or superior performance on visuospatial tests (e.g. ‘physics test’, ‘picture sequencing test’) (see for a review: Baron-Cohen 2009).

The EQ and SQ have shown good cross-cultural stability, see Tables 1 and 2 for an overview of the psychometric properties of the EQ and SQ across different countries. Although the majority of studies have been conducted in the UK (Baron-Cohen and Wheelwright 2004; Baron Cohen et al. 2014; Lawrence et al. 2004; Manson and Winterbottom 2012; Muncer and Ling 2006; Sucksmith et al. 2013; Wheelwright et al. 2006), a large number of studies have validated the EQ by demonstrating the typical sex differences in other European countries (Dimitrijevic et al. 2012; Preti et al. 2011; Vellante et al. 2013; Von Horn et al. 2010; Zeyer et al. 2012), as well as in Canada and the US (Berthoz et al. 2008; Wright and Skagerberg 2012), but to a lesser degree in Asian countries (Kim and Lee 2010; Wakabayashi et al. 2007). The typical sex differences are also present for the SQ in European, Asian as well as US samples (Baron-Cohen et al. 2003; Ling et al. 2009; Manson and Winterbottom 2012; Von Horn et al. 2010; Wakabayashi et al. 2007; Wheelwright et al. 2006; Wright and Skagerberg 2012; Zeyer et al. 2012). Good cross-cultural validity of the measures is also demonstrated by lowered EQ scores and elevated SQ scores in international research on samples of individuals with ASD (Baron-Cohen et al. 2003; Baron-Cohen and Wheelwright 2004; Berthoz et al. 2008; Wakabayashi et al. 2007; Wheelwright et al. 2006).

**Table 1:** Overview of the psychometric properties of the 40-item Empathy Quotient (EQ) across countries

Study (sorted on year of appearance)	Country	Internal consistency (Cronbach’s α)	Test–retest reliability (Pearson r)	N (males)	Females M (SD)	Males M (SD)	Effect size of sex difference (Cohen’s d)
Baron-Cohen and Wheelwright (2004)	UK	0.92	.97^b^	197 (71)	47.2 (10.2)	41.8 (11.2)	0.50
Lawrence et al. (2004)	UK	n.r.	.84^b^	172 (79)	49.6 (9.6)	40.9 (11.9)	0.80
Muncer and Ling (2006)	UK	0.85	n.r.	362 (156)	46.3 (9.5)	37.9 (10.5)	0.84
Wheelwright et al. (2006)	UK	n.r.	n.r.	1761 (723)	48.0 (11.3)	39.0 (11.6)	0.79
Wakabayashi et al. (2007) *control group*	Japan	0.86	n.r.	137 (71)	36.9 (10.7)	31.1 (10.7)	0.54
Wakabayashi et al. (2007) *student group*	Japan	0.86	n.r.	1250 (616)	36.1 (10.4)	30.6 (9.9)	0.54
Berthoz et al. (2008)	Canada (French)	0.81	.93^c^	410 (201)	41.4 (7.7)	37.7 (10.0)	0.41
Kim and Lee (2010)	Korea	0.78	.84^d^	478 (156)	35.8 (9.2)	34.7 (10.5)	0.11
Dimitrijevic et al. (2012)	Serbia	0.78	n.r.	694 (293)	43.1 (9.0)	37.1 (9.4)	0.65
Van Horn et al. (2010)	Sweden	n.r.	n.r.	299 (114)	51.1 (9.7)	43.4 (10.3)	0.78
Preti et al. (2011)	Italy	0.79	.85^d^	256 (118)	45.4 (9.3)	41.8 (9.4)	0.39
Manson and Winterbottom (2012)	UK	n.r.	n.r.	321 (133)	46.4 (12.6)	39.0 (11.7)	0.61
Wright & Skagerberg (2012)^a^	US	0.86-0.87	n.r.	5186 (n.r.)	3.1 (0.30)	2.9 (0.31)	0.66
Zeyer et al. (2012)	Switzerland	0.86	n.r.	500 (250)	43.8 (8.3)	37.7 (10.2)	0.66
Sucksmith et al. (2013)	UK	n.r.	n.r.	187 (93)	48.5 (14.1)	37.7 (13.5)	0.78
Vellante et al. (2013)	Italy	0.80	n.r.	200 (92)	48.3 (8.4)	41.8 (8.7)	0.72
Baron-Cohen et al. (2014)	UK	n.r.	n.r.	3906 (2562)	48.5 (13.7)	38.0 (13.7)	0.76
Present study	Netherlands	0.89	.78^e^	685 (270)	49.0 (10.4)	39.1 (12.0)	0.88

*UK* United Kingdom, *N* sample size, *M* mean, *SD* standard deviation, *n.r.* not reported

^a^The original variant and a reworded variant (with negatively formulated items being rephrased positively) of the EQ were used, and an alternative scoring method was used (mean of the 1, 2, 3, 4 Likert scale rating)

^b^Test–retest period not reported

^c^Test–retest period range 1.5–6 months

^d^Test–retest period of 1 month

^e^Test–retest period of 15 months (range 6–20 months)

**Table 2:** Overview of the psychometric properties of the Systemizing Quotient (SQ) across countries

Study (sorted on year of appearance)	Country	Internal consistency (Cronbach’s α)	Test–retest reliability (Pearson r)	N (males)	Females M (SD)	Males M (SD)	Effect size of sex difference (Cohen’s d)
Baron-Cohen et al. (2003)^a^	UK	0.79	n.r.	278 (114)	24.1 (9.5)	30.3 (11.5)	0.59
Wakabayashi et al. (2007) *control group* ^b^	Japan	0.88	n.r.	137 (71)	17.3 (10.9)	29.5 (10.4)	1.15
Wakabayashi et al. (2007) *student group* ^b^	Japan	0.88	n.r.	1250 (616)	17.7 (9.0)	27.8 (11.8)	0.96
Wheelwright et al. (2006)^c^	UK	0.90	n.r.	1761 (723)	51.7 (19.2)/*(27.7)* ^f^	61.2 (19.2)/*(32.6)* ^f^	0.49
Ling et al. (2009)^a^	UK	0.83	n.r.	167 (84)	22.5 (8.5)	32.1 (10.4)	1.01
Van Horn et al. (2010)^a^	Sweden	n.r.	n.r.	299 (114)	23.9 (8.6)	31.7 (10.4)	0.82
Manson and Winterbottom (2012)^a^	UK	n.r.	n.r.	321 (133)	23.7 (9.6)	33.2 (11.6)	0.89
Wright and Skagerberg (2012)^d^	US	0.91-0.94	n.r.	5186 (?)	2.6 (0.37)	2.8 (0.38)	0.53
Zeyer et al. (2012)^b^	Switzerland	0.83	n.r.	500 (250)	17.7 (10.2)	28.4 (9.0)	1.11
Baron-Cohen et al. (2014)	UK	n.r.	n.r.	3906 (2562)	55.1 (21.1)/*(29.4)* ^*f*^	68.1 (21.6)/*(36.3)* ^*f*^	0.41
Present study	Netherlands	0.87	.79^e^	685 (270)	49.4 (15.3)/*(26.3)* ^*f*^	61.9 (17.9)/*(33.0)* ^*f*^	0.75

*UK* United Kingdom, *N* sample size, *M* mean, *SD* standard deviation

^a^The original 40-item (+20 filler item) version of the SQ was used

^b^A shortened 40-item version of the revised SQ-R was used

^c^The revised 75-item SQ-R was used

^d^The 75-item SQ-R and a reworded variant (with negatively formulated items being rephrased positively) were used, and an alternative scoring method was used (mean of the 1, 2, 3, 4 Likert scale rating)

^e^Test–retest period of 15 months (range 6–20 months)

^f^Recalculated mean score for comparison with the original 40-item SQ (SQ = (SQ-R/75) × 40)

Across international studies, criterion validity of the EQ is indicated by correlations between the EQ and other measures of empathy or measures related to emotional functioning. For example, a strong correlation was found between the EQ and the Friendship Questionnaire, measuring the enjoyment and importance of friendships (Baron-Cohen and Wheelwright 2004), weak to moderate correlations between EQ and both the Interpersonal Reactivity Index measuring affective and cognitive aspects of empathy (Dimitrijevic et al. 2012; Kim and Lee 2010) and the Toronto Alexithymia Scale measuring alexithymia (Preti et al. 2011; Vellante et al. 2013), but only negligible to weak correlations between EQ and the Reading the Mind in the Eyes Test (Vellante et al. 2013). In contrast to the EQ, evidence for cross-cultural validity of the SQ is limited, because only one study outside the UK investigated an ASD sample (Wakabayashi et al. 2007). This study, however, demonstrated good groups validity, because a typical sex difference on the SQ was demonstrated for Japanese participants. Furthermore, Japanese patients with ASD scored higher on the SQ and had more systemizing brain types (as measured by D) compared to the control participants. Only one study investigated the association of SQ with other measures (Ling et al. 2009), and supported its criterion validity by demonstrating that SQ was associated with mental rotation performance and not with general intelligence (Ling et al. 2009).

While several international studies suggest good cross-cultural stability of the EQ and SQ, to date no psychometric properties of a Dutch variant of these questionnaires are available. The aim of the present study was to evaluate the basic psychometric properties of Dutch translations of the EQ and revised version of the SQ (SQ-R) and to investigate whether the mean EQ and SQ-R scores of Dutch males and females are comparable to the scores of other countries as reported in international studies. The SQ-R has previously been created to improve the original SQ by adding more items that might be relevant to females, because the items of the original SQ had primarily been selected from male domains (Wheelwright et al. 2006). Short versions of the EQ have previously been developed containing 28 items (Lawrence et al. 2004) or 15 items (Muncer and Ling 2006). In these short versions, items loading high on social desirability had been removed and factor analyses had demonstrated a clear three-factor structure with the factors Cognitive Empathy (CE), Emotional Empathy (EE), and (Social Skills), that have been partly confirmed in translated versions of the EQ (Berthoz et al. 2008; Dimitrijevic et al. 2012; Preti et al. 2011). Since the questionnaires were developed within the scope of the male brain hypothesis of autism (Baron-Cohen 2009), the groups validity of the questionnaires will be explored by testing for sex differences and ASD-control differences. This study may contribute to the availability of measures for empathizing and systemizing behaviour for Dutch-speaking individuals and moreover to the literature on the cross-cultural stability of the E–S theory of sex differences and autism.

## Methods

### Literature Review

A literature search was performed in PsycInfo and Web of Science to identify international publications on the EQ or SQ. The search terms “EQ”, “Empathy Quotient”, “empathizing”, “SQ”, “Systemizing Quotient” and “systemizing” were used. Only studies written in the English language describing the EQ and/or SQ scores of a healthy sample were included. Studies that only included selected samples, such as patient samples or student samples of specific education types, were excluded. The psychometrics of the identified studies are quantitatively described in Tables 1 and 2, and are discussed in the introduction section. A synthesis is provided in the discussion section. Studies that included an ASD sample in addition to a healthy sample were qualitatively described in the introduction section as well.

### Participants

#### Group 1

Two groups of participants were recruited. *Group 1* is a community sample consisting of 685 adults (270 males, 415 females) in the age of 16–84 years with a mean age of 33 (SD 14.5) years. The participants were recruited via the social networks of the researchers and various psychology students that collaborated on the project, and received no rewards for participation. They were contacted face-to-face, by e-mail or social media with the request to participate and could click a link on the computer to go to the survey. In the survey the participants first read the informed consent of the study and agreed with participation if they continued completing the questionnaire. Subsequently, the participants completed several demographical questions, then the EQ and SQ-R, and finally a questionnaire on symptoms of Attention Deficit Hyperactivity Disorder (this latter questionnaire was not included in the analyses of the current study). The study of *Group 1* was approved by the Ethical Committee of Psychology of the University of Groningen (ppo-011-221, ppo-012-115, test–retest reliability study: ppo-013-077).

Half of the participants were students at the time of the survey (45.4 % fulltime, 5.3 % part time) and the other half were non-students. Half of the participants had a degree in higher professional or academic education (50.6 %), 42.2 % had a diploma in senior secondary vocational/general education or pre-university education, 5.7 % in junior secondary/general education, and 1.2 % finished primary school only (0.3 % had missing data). A quarter of the sample had a fulltime job, 40.0 % a part time job and 34.7 % had no occupation at the time of the study, and 0.3 % had missing data. One third of the sample was single (32.1 %), one third was married (28.9 %) or had a registered partnership (2.0 %), and one-third had a partner with whom they were living together (11.5 %) or living apart together (22.8 %), and a minority was divorced (2.0 %) or widower/widower (0.6 %). In the sample, 5.1 % indicated that they were diagnosed with a mental disorder and 2.8 % indicated using medication for mental complaints.

For the calculation of the test–retest reliability, 164 participants who had left their e-mail address for a follow-up study, were asked to complete the EQ and SQ-R again. In total 58 participants (22 males, 36 females) completed both questionnaires for a second time, with an average time between test and retest of 15 months (ranging from 6 to 20 months). Nineteen participants were students at the time of the study, and of the 58 participants 40 had a part-time or fulltime job and the remaining 18 had no job. More than half of the participants (n = 36) had a degree in higher professional or academic education. Three participants indicated that they were diagnosed with a mental disorder and two used medication for mental complaints.

#### Group 2

*Group 2* consisted of 42 males with a formal clinical diagnosis in the autistic spectrum in the age of 17–34 and a mean age of 22 (SD 4.2) years. The participants were recruited from the outpatient clinic of Accare, i.e. the University Child and Adolescent Psychiatry Center, Groningen, and from the Autism Team of the Northern Netherlands, Jonx/Lentis, Groningen. All patients had been assessed for the presence of an ASD according to the DSM-IV criteria by at least one experienced clinician, who was not involved in this study. Patients had to meet the criteria for either Autism Disorder, Asperger’s Syndrome or Pervasive Developmental Disorder Not Otherwise Specified. The assessment was performed by extensive (hetero)anamnestic interviews. For 12 cases the Autism Diagnostic Observation Scale (ADOS) (Lord et al. 2000) had been performed during the clinical assessment, and all 12 cases obtained clinical scores on at least one of its subscales. More specifically, 7 patients scored above the clinical cut-off on the Communication Scale (2+), 10 patients scored above the clinical cut-off on the Social Interaction Scale (4+), and 3 patients scored in the clinical range on both scales. For the remaining 30 patients no gold standard diagnostic measure for autism was available at the time of data-collection. These patients were described by means of the AQ and SRS-A (see “Materials” section). Moreover, patients were only included when the clinician judged the patients as having an intelligence level within the normal range (IQ ≥ 80). In case of doubt, patients performed a short version of the Groninger Intelligence Test (GIT) (Luteijn and Barelds 2004). For 19 cases the GIT was administered, and this subgroup scored in the range of 80–128 with an average IQ of 103 (SD 15.3). The patients completed paper-and-pencil versions of the EQ and SQ-R as part of a (pilot for a) treatment study which was approved by the Medical Ethical Committee of the University Medical Center Groningen (METc 2010.133).

### Materials

#### EQ (Group 1 and 2)

The original 40-item EQ plus 20 filler items (Baron-Cohen and Wheelwright 2004) was translated into Dutch by the author YG and the translation was checked by the author AdH (the Dutch EQ, scoring key, and norm table can be requested from the corresponding author). The EQ items are rated on a 4-point Likert-scale (strongly agree, slightly agree, slightly disagree, strongly disagree). The 20 filler items (2, 3, 5, 7, 9, 13, 16, 17, 20, 23, 24, 30, 31, 33, 40, 45, 47, 51, 53, 56) are not counted in the scoring. A three point scoring system was adopted from Baron-Cohen and Wheelwright (2004), discriminating ‘lacking’, ‘mildly’ and ‘strongly’ empathic behaviour. The 21 forward items (1, 6, 19, 22, 25, 26, 35, 36, 37, 38, 41, 42, 43, 44, 52, 54, 55, 57, 58, 59, 60) are scored 2 for ‘strongly agree’, 1 for ‘slightly agree’, and 0 for ‘strongly disagree’ and ‘slightly disagree’. The 19 reversed items (4, 8, 10, 11, 12, 14, 15, 18, 21, 27, 28, 29, 32, 34, 39, 46, 48, 49, 50) are scored 2 for ‘strongly disagree’ and 1 for ‘slightly disagree’, and 0 for ‘strongly agree’ and ‘slightly agree’. Previous factor-analytic studies distinguished three EQ subscales labelled ‘Cognitive Empathy’ (CE), ‘Emotional Empathy’ (EE), and ‘Social Skills’ (SS), either based on 28 items (Lawrence et al. 2004) or on 15 items (Muncer and Ling 2006). See Table 3 for an overview of the items belonging to each subscale.

**Table 3:** Distribution of the 40 items of the Emotional Quotient (EQ) across the three subscales

Item	28-item version	15-item version
1. I can easily tell if someone else wants to enter a conversation	CE	
4. I find it difficult to explain to others things that I understand easily, when they don’t understand it first time (R)	SS	SS
6. I really enjoy caring for other people	EE	EE
8. I find it hard to know what to do in a social situation (R)	SS	SS
10. People often tell me that I went too far in driving my point home in a discussion (R)		
11. It doesn’t bother me too much if I am late meeting a friend (R)		
12. Friendships and relationships are just too difficult, so I tend not to bother with them (R)	SS	SS
14. I often find it difficult to judge if something is rude or polite (R)	SS	SS
15. In a conversation, I tend to focus on my own thoughts rather than on what my listener might be thinking (R)		
18. When I was a child, I enjoyed cutting up worms to see what would happen (R)		
19. I can pick up quickly if someone says one thing but means another	CE	
21. It is hard for me to see why some things upset people so much (R)	EE	
22. I find it easy to put myself in somebody else’s shoes	EE	
25. I am good at predicting how someone will feel	CE	CE
26. I am quick to spot when someone in a group is feeling awkward or uncomfortable	CE	CE
27. If I say something that someone else is offended by, I think that that’s their problem, not mine (R)	EE	EE
28. If anyone asked me if I like their haircut, I would reply truthfully, even if I didn’t like it (R)		
29. I can’t always see why someone should have felt offended by a remark (R)	EE	
32. Seeing people cry doesn’t really upset me (R)	EE	EE
34. I am very blunt, which some people take to be rudeness, even though this is unintentional (R)		
35. I don’t tend to find social situations confusing	SS	SS
36. Other people tell me I am good at understanding how they are feeling and what they are thinking	CE	
37. When I talk to people, I tend to talk about their experiences rather than my own		
38. It upsets me to see animals in pain		
39. I am able to make decisions without being influenced by people’s feelings (R)		
41. I can easily tell if someone else is interested or bored with what I am saying	CE	
42. I get upset if I see people suffering on news programmes	EE	
43. Friends usually talk to me about their problems as they say I am very understanding	EE	
44. I can sense if I am intruding, even if the other person doesn’t tell me	CE	CE
46. People sometimes tell me that I have gone too far with teasing (R)		
48. Other people often say that I am insensitive, though I don’t always see why (R)	EE	
49. If I see a stranger in a group, I think that it is up to them to make an effort to join in (R)		
50. I usually stay emotionally detached when watching a film (R)	EE	EE
52. I can tune into how someone else feels rapidly and intuitively	CE	CE
54. I can easily work out what another person might want to talk about	CE	CE
55. I can tell if someone is masking their true emotion	CE	
57. I don’t consciously work out the rules of social situations	SS	
58. I am good at predicting what someone will do	CE	
59. I tend to get emotionally involved with a friend’s problems	EE	EE
60. I can usually appreciate the other person’s viewpoint, even if I don’t agree with it		

The number in front of item represents the position in original 40-item questionnaire that was completed with 20 filler items (adding up to 60 items in total). The filler items are not depicted in this table

*R* Reverse keyed item, *CE* Cognitive Empathy subscale, *EE* Emotional Empathy subscale, *SS* Social Skills subscale

#### SQ-R (Group 1 and 2)

The revised version of the SQ (SQ-R) (Wheelwright et al. 2006) was translated into Dutch by the author YG and the translation was checked by the author AdH (the Dutch SQ-R, scoring key, and norm table can be requested from the corresponding author). The 75 items are rated on a 4-point Likert-scale (strongly agree, slightly agree, slightly disagree, strongly disagree). A three point scoring system was adopted from Wheelwright et al. (2006), discriminating ‘lacking’, ‘mildly’ and ‘strongly’ systemizing behaviour. The 36 reversed items (3, 6, 8, 10, 15, 17, 22, 24, 26, 28, 31, 33, 34, 35, 37, 39, 40, 44, 45, 47, 48, 49, 51, 52, 54, 56, 57, 58, 59, 63, 64, 65, 67, 70, 71, 73) are scored 2 for ‘strongly disagree’ and 1 for ‘slightly disagree’, and 0 for ‘strongly agree’ and ‘slightly agree’. In contrast, the remaining 39 forward items are scored 2 for ‘strongly agree’ and 1 for ‘slightly agree’, and 0 for ‘strongly disagree’ and ‘slightly disagree’.

#### Brain Type: D (Group 1 and 2)

Based on the EQ and SQ-R scores the participant’s brain type can be calculated. For this calculation, first the EQ and SQ-R total scores were standardized by the estimated population means of *Group 1* (n = 685), using the formulas E = [EQ − M(EQ)/(maximum possible score)] and S = [SQ-R − M(SQ-R)/(maximum possible score)]. A continuous measure for brain type is calculated by the formula D = [(S − E)/2] (see Wheelwright et al. 2006). A positive score on D indicates brain Type S, or Extreme Type S, a negative score indicates brain Type E, or Extreme Type E, and a score close to zero indicates brain Type B.

#### FQ (Group 1)

The Friendship Questionnaire (FQ) is a 35-item self-report questionnaire measuring a person’s enjoyment and importance of friendships and interest in other people (Baron-Cohen and Wheelwright 2003), translated by Uzieblo, De Corte, Crombez, and Buysse (unpublished). On each item, the participants have to decide which statement about friendships and social interactions is most applicable to them. On each item two, three, four or five statements are presented. For example, on item 1 the participant has to choose between the following statements: “I have one or two particular best friends”; “I have several friends who I would call best friends”; “I don’t have anybody who I would call a best friend”. Twenty-seven out of 35 items are included in the scoring with a maximum score of 5 per item, resulting in a maximum total score of 135. Approximately half of the items are reverse keyed items. The FQ was demonstrated to have high internal consistency (Cronbach’s α = 0.84) in a mixed ASD and healthy control sample, and good criterion validity as demonstrated by large sex differences (females scoring higher than males) and individuals with ASD scoring lower than healthy controls with large effect size (Baron-Cohen and Wheelwright 2003).

#### SRS-A (Group 2)

The Social Responsiveness Scale-Adults (SRS-A) (Noens et al. 2012) is a scale that can be used as both a screening test and as an aid to clinical diagnosis for ASD (Aldridge et al. 2012). It consists of 64 items covering the various dimensions of interpersonal behaviour, communication and repetitive/stereotypic behaviours that are typical for ASD, and is rated on a 4-point Likert scale (not true, sometimes true, often true, almost always true). For 20 patients the informant version of the SRS-A was available which was completed by a close relative or friend (mean score 77; SD 32; range 31–147). Of this subgroup, 13 patients scored above the clinical cut-off score of 61.

#### AQ (Group 2)

The Dutch translation of the Autism Spectrum Quotient (AQ) (Hoekstra et al. 2008) was administered in 40 patients in order to describe their self-experienced autistic traits. The AQ consists of 50 items assessing personal preferences and habits related to ASD, and is rated on a 4-point Likert scale (definitely agree, slightly agree, slightly disagree, definitely agree). Half of the items are reverse keyed. The British scoring method with dichotomized answer categories (agree, disagree) was used (Baron-Cohen et al. 2001).

For 40 of the 42 patients an AQ was available and this group obtained a mean score of 25 (SD 7.7; range 9–40). Only 8 of them scored above the clinical cut-off score for the British population of 32+. Interestingly, 15 out of the 17 patients who scored beneath this AQ cut-off score and for whom also an ADOS or SRS-A score was available obtained a clinical score on the ADOS or SRS-A. This confirms that most of the patients in this study reporting subclinical AQ scores still show problems in the autistic spectrum according to their friends, families or professionals. In comparison to the other clinical ratings, low AQ scores may be a consequence of the patients’ impairment in self-reflection and awareness of their autistic traits which has previously been observed in patients with Asperger syndrome (Jackson et al. 2012). An underestimation of autistic traits has also been observed in children/adolescents with ASD, as compared to parent ratings (Johnson et al. 2009).

### Statistical Analyses

#### Data Cleaning

All analyses, except for the factor analyses, were performed using SPSS 20 (IBM Corp.). Completion of the SQ-R (75 items) and EQ (40 items) by a large number of participants (n = 691) went along with few missing values that were due to participants occasionally omitting to provide an answer to a question. For missing values an imputation model was used (including all variables of the respective scale) that was estimated by maximum likelihood (ML), obtaining a singly imputed data set. One respondent omitted to complete the SQ-R and was therefore excluded. Five participants were excluded from further analysis. They were suspected for careless completion of the questionnaires, because they filled out scores on the FQ items 30 and 34 that were not credible. Because of an error in the online survey, part of the participants omitted to provide an answer to the FQ item 30 (n = 161), item 34 (n = 155), and item 25 (n = 381). These items were also imputed using a model that was estimated by using ML. Data of 685 respondents entered the final analysis.

#### Confirmatory Factor Analyses (CFA)

Factor analysis was performed in LISREL 8.8 (Jöreskog and Sörbom 2006) in order to determine whether the three-factorial structure of the EQ and the one-factorial structure of the SQ-R could be replicated in this Dutch sample. With regard to the EQ, separate confirmatory factor analyses (CFA) were performed on the 28-item version (Lawrence et al. 2004) and the 15-item version (Muncer and Ling 2006) in order to test whether the data fitted the previously proposed three-factor structure. The following models were tested: (1) three-factor model competing with a one-factor model of the 28-item version, (2) three-factor model competing with a one-factor model of the 15-item version, (3) one-factor model of the 40-item version without competing models. The original 40 items of the EQ as well as the item distributions across the three factors (CE, EE, and SS) for both the 15-item version and the 28-item version are presented in Table 3. The three-factor models were only tested for the short-versions, because no factor structure had previously been proposed for the EQ containing all original 40 items, and some of the items load strongly on social desirability (Berthoz et al. 2008; Dimitrijevic et al. 2012; Preti et al. 2011). Diagonally Weighted Least Square (DWLS) estimation method was applied for all CFAs because of an ordered-categorical response format. Scaling of latent variables was achieved by setting the factor variance to 1. The t-rule was applied for identification of latent variables (Bollen 1989). All analyses were carried out on the total sample of healthy participants (n = 685) which considerably exceeds the criterion of a minimum sample size of 200 respondents for CFA (Hinkin 1998).

The fit of the respective factor structure was evaluated by the following statistics of CFA: Chi-Square value with corresponding *p* value, normed Chi-Square (χ^2^/*df*), Root Mean Squared Error of Approximation (RMSEA), 90 %-confidence interval (CI) of the RMSEA, Standardized Root Mean Square Residual (SRMR) and Comparative Fit Index (CFI). The *Chi*-*Square value* with its corresponding *p* value belongs to the class of absolute fit indices (Hu and Bentler 1999). Disadvantages of Chi-Square statistics are that both deviations from normality and large sample sizes may result in model rejection (Hooper et al. 2008). Therefore, less weight was given to the Chi-Square test than to the descriptive measure of the *normed Chi*-*Square* (Wheaton et al. 1977). Recommendations for an acceptable ratio of the *normed Chi*-*Square* range from 5.0 to 2.0 with a *good fit* below a value of 3.0 (Hinkin 1998; Hooper et al. 2008). Furthermore, the *Root Mean Squared Error of Approximation* (RMSEA) and a 90 %-CI of the RMSEA were calculated (Steiger 1990). There is consensus about an upper limit of RMSEA of 0.07 (Steiger 2007) and of an upper limit of the CI of the RMSEA of less than 0.08 (Hooper et al. 2008). The *Standardized Root Mean Square Residual* (SRMR) ranges from 0 to 1 with acceptable models obtaining values up to 0.08 (Hu and Bentler 1999). The *Comparative Fit Index* (CFI) is a revised version of the Non-Normed Fit Index (NNFI), also known as Tucker-Lewis Index (Bentler 1990). There is an agreement that a CFI of ≥0.90 to ≥0.95 indicates a good model fit (Hu and Bentler 1999). The goodness-of-fit statistics of the respective factor model were compared to the cut-offs and recommendations as cited above.

#### Exploratory Factor Analysis (EFA)

With regard to the SQ-R, exploratory factor analysis was conducted by using principal axis factoring (PAF) with oblique rotation method to test whether there are several statistical meaningful clusters of items representing psychologically meaningful concepts. Parallel analysis was performed to determine the number of factors to retain in PAF. In parallel analysis (PA-PAF), random data matrices of the same size as the actual data set were generated and eigenvalues were computed for the correlation matrices of each of the random data sets. Eigenvalues of random data sets and actual data sets were compared and the number of factors to retain was determined by those factors whose eigenvalues in the random data set exceeded the eigenvalues in the actual data set. A scree plot inspection was additionally performed to support factor retention criterion in PA-PAF.

#### Reliability

The internal consistency of the SQ-R, the EQ scale and subscales was estimated using Cronbach’s α. The test–retest reliability was computed for the SQ-R and the EQ scales by Pearson correlations. Furthermore, reliability of the continuous measure for brain type (D) was derived. As the index D is a difference score of standardized EQ and SQ-R scores, its reliability was estimated by taking the reliabilities of EQ and SQ-R into consideration and controlling for the observed score correlation between both measures (Kessler 1977; Linn and Slinde 1977; Rogosa and Willett 1983).

#### Validity

For validation of the EQ and SQ-R, sex differences were tested on the EQ, SQ-R, and D. It was hypothesized that females obtain higher scores on EQ and lower scores on SQ-R and D, i.e. showing a more empathizing brain type. Additionally, it was tested whether males with ASD compared to control males score lower on EQ and higher on SQ-R, and D, i.e. showing a more systemizing brain type. For this purpose, means and standard deviations of the EQ scales, SQ-R, and D (the continuous measure of brain type) were calculated for males and females of *Group 1*, and also for *Group 2* (the males with ASD). In order to explore groups validity, independent-samples t-tests were used to estimate sex differences in *Group 1* and differences between the ASD patients in *Group 2* and the males of *Group 1* on the EQ scales, the SQ-R, and D. Effect sizes (Cohen’s d) were calculated for all comparisons to indicate the magnitude of group differences. Further calculations were performed on the EQ version with the best psychometric properties (15-item, 28-item version or 40-item version). To further explore criterion validity, in *Group 1 and 2* correlational analysis between the SQ-R, the EQ scales, D, and the FQ (only in group 1) or the AQ (only in group 2) was performed, using Pearson’s correlation coefficients. The correlational analyses were separated for the two groups in order to explore differential correlational patterns in the ASD group compared to the typical group, e.g. the strength of the trade-off between EQ and SQ that has previously been suggested to be higher in people with ASD (Wheelwright et al. 2006).

#### Classification Statistics

A receiver operating characteristic (ROC) analysis was conducted in order to explore the accuracy of SQ-R, EQ and D in detecting males with ASD relative to healthy males of *Group 1*. A ROC analysis distinguishes between true positive rates and true negative rates. Whereas the true positive rate (sensitivity) describes the proportion of individuals with autism who are correctly identified as having the condition, the true negative rate (specificity) describes the proportion of healthy individuals who are correctly identified as not having the condition. A ROC curve plots the sensitivity against ‘1—specificity’ at each level of the scale under scrutiny (i.e. SQ-R, EQ or D) to predict the criterion (distinguishing healthy males from males with ASD). ROC analysis allows for determination of an overall accuracy of classification as measured by the area under the curve (AUC), as well as classification statistics to address specific goals, i.e. high sensitivity or high specificity.

## Results

### Factor Structure and Reliability of the EQ and SQ-R

#### Three-Factor Structure of the EQ and Reliability

Table 4 presents all item loadings and goodness-of-fit statistics of the CFAs applied to the 40-item version (one-factor model), 28-item version (one-factor and three-factor model) and 15-item version (one-factor and three factor model) of the EQ. Overall, CFA supported the previously proposed three-factor structure of the EQ in both the 28-item version and the 15-item version. Item loadings of the three-factor models ranged from 0.10 to 0.83 (28-item version) and from 0.36 to 0.85 (15-item version). All item loadings of the three-factor models (both 28-item and 15-item version) were ≥0.30 with the exception of the loading of item 57 in the three-factor model of the 28-item version. Item loadings of the one-factor models ranged from 0.03 to 0.79 (40-item version), from 0.07 to 0.80 (28-item version), and from 0.28 to 0.80 (15-item version). Whereas the one-factor model of the 15-item version contained only one item with a loading of <0.30 (item 4), the one-factor model of the 28-item version contained two of such items (item 4 and 57) and the one-factor model of the 40-item version contained 7 items with a loading of <0.30 (items 4, 57, 10, 11, 18, 28, and 39). The goodness-of-fit statistics of the three factor-model on the 28-item as well as 15-item version clearly outperformed the respective competing one-factor models (28-item and 15-item version) (see Table 4).

**Table 4:** Item loadings and goodness-of-fit statistics of the confirmatory factor analysis (CFA) of the EQ

Item number	Three-factor (28-item)	Three-factor (15-item)	One-factor (40-item)	One-factor (28-item)	One-factor (15-item)
1.	0.70 (CE)		0.68	0.68	
19.	0.61 (CE)		0.56	0.58	
25.	0.77 (CE)	0.78 (CE)	0.72	0.75	0.73
26.	0.82 (CE)	0.85 (CE)	0.77	0.79	0.80
36.	0.80 (CE)		0.77	0.77	
41.	0.67 (CE)		0.64	0.59	
44.	0.75 (CE)	0.72 (CE)	0.71	0.72	0.68
52.	0.83 (CE)	0.82 (CE)	0.79	0.80	0.78
54.	0.77 (CE)	0.76 (CE)	0.72	0.74	0.71
55.	0.72 (CE)		0.67	0.69	
58.	0.65 (CE)		0.59	0.62	
6.	0.54 (EE)	0.63 (EE)	0.49	0.48	0.48
21.	0.73 (EE)		0.66	0.65	
22.	0.77 (EE)		0.69	0.69	
27.	0.35 (EE)	0.47 (EE)	0.34	0.30	0.32
29.	0.49 (EE)		0.47	0.44	
32.	0.56 (EE)	0.75 (EE)	0.51	0.48	0.50
42.	0.40 (EE)		0.39	0.35	
43.	0.79 (EE)		0.71	0.71	
48.	0.67 (EE)		0.63	0.58	
50.	0.45 (EE)	0.57 (EE)	0.41	0.39	0.39
59.	0.50 (EE)	0.64 (EE)	0.46	0.44	0.44
4.	0.34 (SS)	0.36 (SS)	0.25	0.25	0.28
8.	0.69 (SS)	0.70 (SS)	0.47	0.50	0.54
12.	0.65 (SS)	0.67 (SS)	0.49	0.48	0.54
14.	0.62 (SS)	0.53 (SS)	0.48	0.46	0.43
35.	0.67 (SS)	0.69 (SS)	0.47	0.49	0.53
57.	0.10 (SS)		0.07	0.07	
10.			0.25		
11.			0.13		
15.			0.36		
18.			0.25		
28.			0.06		
34.			0.30		
37.			0.33		
38.			0.36		
39.			0.03		
46.			0.35		
49.			0.25		
60.			0.48		
Fit statistics					
χ^2^ (*df*)^a^	1192 (347)	197 (87)	10,117 (740)	2162 (350)	774 (90)
*p*	<.001	<.001	<.001	<.001	<.001
χ^2^/*df*	3.44	2.26	13.67	6.18	8.60
RMSEA	0.060	0.043	0.088	0.087	0.11
CI-RMSEA	0.056;0.063	0.035;0.051	0.086;0.091	0.083;0.090	0.099;0.11
SRMR	0.077	0.058	0.099	0.092	0.10
CFI	0.97	0.99	0.90	0.94	0.91

*CE* Cognitive Empathy subscale, *EE* Emotional Empathy subscale, *SS* Social Skills subscale, *RMSEA* Root Mean Squared Error of Approximation, *CI-RMSEA* 90 % confidence interval of RMSEA, *SRMR* Standardized Root Mean Square Residual, *CFI* Comparative Fit Index

^a^Satorra–Bentler Scaled Chi-Square

The internal consistency of the 40-item EQ was good (Cronbach’s α = 0.89). The overall scale reliability of the 28-item EQ was also good (Cronbach’s α = 0.89), as well as the reliability of its subscales CE (Cronbach’s α = 0.89) and EE (Cronbach’s α = 0.80). The SS subscale, however, had moderate reliability (Cronbach’s α = 0.57). Note that reversed items had slightly lower reliability (Cronbach’s α = 0.74) than forward items (Cronbach’s α = 0.88), and that the CE scale did not contain any reversed items. The EE scale consisted for half of reversed items (6 out of 11), and the SS scale consisted mainly of reversed items (4 out of 6), see Table 3. Lower reliability of the reversed items might hence have influenced the lower internal consistency of the SS scale. The overall scale reliability of the 15-item version of the EQ was also good but lower than the 28-item version (Cronbach’s α = 0.80). The 15-item subscales were also less reliable compared to the 28-item version, with a good reliability of the CE scale (Cronbach’s α = 0.83), but questionable reliability of the EE (Cronbach’s α = 0.67) and SS (Cronbach’s α = 0.62) subscales. Based on the previous analyses we preferred the 28-item EQ over the 40-item and 15-item EQ, because this version provides better (sub)scale reliability. The 28-item version was therefore used in further analyses.

The test–retest reliability was good (40-item EQ: r(58) = 0.78, *p* < .001; 28-item EQ: r(58) = 0.74, *p* < .001). Furthermore, the test–retest reliability of the 28-item EQ subscales CE and SS was also good (EQ CE: r(58) = 0.74, *p* < .001; EQ SS: r(58) = 0.76, *p* < .001), although the EE scale was somewhat less reliable (EQ EE: r(58) = 0.58, *p* < .001).

#### Factor Structure of the SQ-R and Reliability

Exploratory factor analysis (principal axis factoring (PAF) with oblique rotation method) on the SQ-R failed to demonstrate statistical meaningful clusters of items. PAF extracted 8 factors with an eigenvalue of greater than 1, explaining together 28.2 % of the total variance. Parallel analysis (PA-PAF) and scree plot inspection was performed in order to determine the number of factors to retain in PAF, resulting in 5 factors to retain. However, the large number of factors to retain could partly be explained by the chosen analysis technique, given the tendency of PA-PAF towards over-extraction (which might also affect the psychological interpretation of factors). The five factors retained in PAF explained 23.3 % of the total variance, with 10.6 % explained by factor 1 (eigenvalue 7.9), 4.4 % explained by factor 2 (eigenvalue 3.3), 3.5 % explained by factor 3 (eigenvalue 2.6), 2.6 % explained by factor 4 (eigenvalue 2.0) and 2.2 % explained by factor 5 (eigenvalue 1.7). An examination of the items in each factor did not reveal psychologically meaningful clusters, and therefore a single factor model was adopted.

Internal consistency (scale reliability) for the single factor model was good (Cronbach’s α = 0.87) and did not improve significantly if single items were excluded (Cronbach’s α ranged between 0.869 and 0.875 if single items were deleted). Note that the 36 reversed items had slightly lower reliability (Cronbach’s α = 0.77) than the 39 forward items (Cronbach’s α = 0.82). The test–retest reliability of the SQ-R was good (r(58) = 0.79, *p* < .001). The test–retest reliability of D (as determined by reliabilities of EQ and SQ-R as well as by correcting for the correlation of EQ and SQ-R) was also good (r = 0.78). As the EQ correlated only weakly with the SQ-R (r(58) = −0.10, *p* < .01), the corrected reliability of D was only slightly lowered compared to the uncorrected reliability.

### Validity of the EQ and SQ-R

#### Groups Validity

Means and standard deviations of the 28-item EQ, the SQ-R and brain type D for *Group 1* and *Group 2* are presented in Table 5. The expected sex differences were found, as the females showed significantly higher scores on the EQ than males with medium effect size. Large sex differences were found on the subscale EE, whereas small sex differences were present for the subscales CE and SS. In line with the expectations females showed significantly lower scores on the SQ-R compared to males with medium effect size. Also in line with the expectations, D was significantly lower in females compared to males with large effect size, indicating more empathic brain types in females.

**Table 5:** Means and standard deviations of the 28-item EQ, the 75-item SQ-R and ‘brain type’ (D) for *Group 1* (healthy sample) and *Group 2* (ASD sample)

	Group 1		Group 2	Group comparisons					
	Females (n = 415)	Males (n = 270)	ASD males (n = 42)	Females versus males (*df* = 683)			Males versus ASD males (*df* = 310)		
	M ± SD	M ± SD	M ± SD	t	*p*	d	t	*p*	d
EQ-total (28-items)	35.9 ± 8.6	28.8 ± 9.8	18.5 ± 9.6	10.02	<.001	0.77	6.26	<.001	1.06
EQ–CE	14.1 ± 4.7	11.9 ± 4.8	7.5 ± 5.0	5.96	<.001	0.46	5.44	<.001	0.90
EQ–EE	15.9 ± 4.1	11.4 ± 4.7	8.4 ± 4.4	13.4	<.001	1.02	3.78	<.001	0.66
EQ–SS	7.5 ± 2.4	6.9 ± 2.6	3.6 ± 2.6	3.04	.002	0.24	7.56	<.001	1.27
SQ-R (75 items)	49.4 ± 15.3	61.9 ± 17.9	59.8 ± 20.0	−9.79	<.001	0.75	0.71	.480	0.11
D	−0.041 ± 0.089	0.064 ± 0.107	0.147 ± 0.093	−13.95	<.001	1.07	−4.79	<.001	0.83

*CE* Cognitive Empathy, *EE* Emotional Empathy, *SS* Social Skills, *D* difference between standardized EQ and SQ (‘brain type’)

The expected ASD-control differences were found as well. As can be seen in Table 6, the males with ASD showed significantly lower scores on all EQ scales than the male participants of *Group 1* (the norm group). All scales showed large effect sizes for these group differences, except for the EE subscale, which showed a medium effect. The males with ASD did not differ significantly from the males of *Group 1* on the SQ-R with a negligible effect size. They, however, differed with large effect size on D, indicating a more positive score, i.e. a more systemizing brain type, for males with ASD compared to the norm group.

**Table 6:** Correlations in *Group 1* (healthy sample, n = 685) between the 28-item Empathy Quotient (EQ) scales, Systemizing Quotient-Revised (SQ-R), ‘brain type’ (D) and Friendship Quotient (FQ)

	EQ–CE	EQ–EE	EQ–SS	SQ-R	D	FQ
EQ-total	0.873***	0.871***	0.657***	−0.102**	−0.849***	0.434***
EQ–CE	–	0.604***	0.432***	−0.007	−0.697***	0.268***
EQ–EE		–	0.404***	−0.168***	−0.781***	0.503***
EQ–SS			–	−0.066	−0.557***	0.257***
SQ-R				–	0.613***	−0.231***
D					–	−0.467***

*CE* Cognitive Empathy, *EE* Emotional Empathy, *SS* Social Skills, *D* difference between standardized EQ and SQ (‘brain type’)

* *p* < .05; ** *p* < .01; *** *p* < .001

#### Criterion Validity

Table 6 shows an overview of the intercorrelations of the 28-item EQ scales and the correlations between the FQ, EQ, SQ-R and D scores in *Group 1*. Correlational analysis revealed significant strong intercorrelations of the EQ total scale and the subscales, with the exception that the SS subscale showed significant moderate intercorrelations with the CE and EE subscales. The intercorrelations of the 15-item EQ scales were smaller compared to the 28-item version, ranging from weak (EE and SS: r = 0.282, *p* < .001) to moderate (CE and EE: r = 0.345, *p* < .001; CE and SS: r = 0.438, *p* < .001). The FQ was found to be positively correlated with all EQ scales (weak to moderate associations), to be negatively correlated with the SQ-R (weak association), and to be negatively correlated with D (moderate association). Exploring associations between the SQ-R and EQ scales revealed a significant weak negative correlation between the SQ-R and the EE subscale and nonsignificant negligible correlations with the other subscales. Consequently, the total EQ scale correlated negatively and only weakly with SQ-R. Strong negative correlations were present between D and all EQ scales and a strong positive correlation with SQ-R. The strong correlations between D and the EQ scales and D and the SQ-R are likely caused by D being a composite score of EQ and SQ-R.

As can be seen in Table 7, in contrast to *Group 1*, the correlations between the SQ-R and EQ scales in the ASD group were positive and had moderate to strong strength. The correlations of D with all EQ scales and SQ-R in the ASD group were similar to *Group 1*. AQ in the ASD group showed moderate to strong negative correlations with all EQ scales and a strong positive correlation with D, but a non-significant positive correlation with SQ-R.

**Table 7:** Correlations in *Group 2* (ASD sample, n = 42) between the 28-item Empathy Quotient (EQ) scales, Systemizing Quotient-Revised (SQ-R), ‘brain type’ (D) and Autism spectrum Quotient (AQ)

	EQ–CE	EQ–EE	EQ–SS	SQ-R	D	AQ^a^
EQ-total	0.879**	0.828**	0.773**	0.282	−0.723**	−0.603**
EQ–CE	–	0.532**	0.629**	0.358*	−0.556**	−0.511**
EQ–EE		–	0.455**	0.229	−0.602**	−0.372*
EQ–SS			–	0.014	−0.706**	−0.744**
SQ-R				–	0.459**	0.172
D					–	0.686**

*CE* Cognitive Empathy, *EE* Emotional Empathy, *SS* Social Skills, *D* difference between standardized EQ and SQ (‘brain type’)

* *p* < .05; ** *p* < .01; *** *p* < .001

^a^n = 40 due to 2 missing values

#### Predictive Validity

The accuracy of SQ-R, EQ and D in detecting males with ASD (n = 42) relative to healthy males (n = 270) was examined by means of ROC analyses. Classification statistics indicating sensitivity, specificity, Positive Predictive Value (PPV) and Negative Predictive Value (NPV) for various cut-offs are presented in Table 8. A significantly higher accuracy than chance of detecting males with ASD was revealed for the EQ (AUC = 0.766; SE = 0.039; *p* < .001) and D (AUC = 0.740; SE = 0.039; *p* < .001). For both EQ and D, the specificity at a cut-off score of *p* = 97.5 and *p* = 2.5 respectively was higher than 0.90, meaning that participants with extremely high EQ scores or extremely low D scores (thus extreme empathizing brain types) can be classified quite accurately as not having ASD. Sensitivity was however insufficient, meaning that participants with low EQ scores and high D scores (thus systemizing or extreme systemizing brain types) cannot be accurately classified as having ASD. The combinations of sensitivity and specificity can therefore be regarded as suboptimal in the detection of males with ASD. No predictive validity was yielded for the SQ-R (AUC = 0.547; SE = 0.052; *p* = .331). At various cut-offs the combinations of sensitivity and specificity were poor, meaning that SQ-R scores cannot accurately classify participants as having ASD or not.

**Table 8:** Classification accuracy of EQ (28 item version), SQ-R and D in detecting males with ASD (n = 42) relative to healthy males (n = 270)

	Sensitivity	Specificity	PPV	NPV
28-item EQ				
Perc ≤ 2.5	40.5	94.5	53.1	91.1
Perc ≤ 35	80.1	45.9	18.9	93.9
SQ-R				
Perc ≥ 97.5	2.4	95.2	7.1	86.2
Perc ≥ 65	42.9	47.0	11.2	84.1
D				
Perc ≥ 97.5^a^	9.5	97.0	33.3	87.3
Perc ≥ 65^b^	83.3	38.9	17.5	93.8

Cut-off criteria were applied to the indicated percentiles

*PPV* Positive Predictive Value, *NPV* Negative Predictive Value

^a^Extreme systemizing brain type

^b^Systemizing brain type and Extreme systemizing brain type

## Discussion

The aim of this study was to describe the psychometric properties of Dutch translations of the EQ and SQ-R questionnaires and to review the cross-cultural validity of the EQ and SQ. To this end the reliability and validity of the original 40-item EQ, the short versions of the EQ (15- and 28-item versions), and the SQ-R were tested in a Dutch-speaking healthy sample and a patient sample of males with ASD. The psychometric properties of the Dutch EQ and SQ-R of the healthy sample were compared to the psychometric properties described in the international literature. For this purpose, the international studies on the EQ and SQ had been systematically reviewed in the introduction section and a synthesis on the cross-cultural validity of the EQ and SQ is explicated below.

The EQ mean scores of the Dutch sample reported in the present study are comparable to the scores of other Western countries (see Table 1). The sex differences are also comparable in magnitude, with medium effect size, as compared to the medium to large effect sizes of sex differences in the other countries. Reviewing the current literature on the EQ revealed that the average EQ scores of both males and females in Asian countries (for both student and community samples) are roughly one standard deviation lower compared to Western countries, and also the sex differences in these Asian countries are only small in effect size (and not always significant for the total EQ scale). This may be explained by cultural differences in the emotional and social habits of people in Western and Asian countries, e.g. in Western countries it is much more desired to openly express one’s emotions than in Asian countries (Eid and Diener 2001). In Asian countries, empathy may therefore be expressed to a lesser extent in social situations, and sex differences in the inner emotional life may therefore be underestimated or less well recognized when completing the EQ. Concerning cross-cultural stability of the EQ, it can be concluded that findings are stable in Western countries, but that EQ is characterised by a lower stability and sensitivity for sex differences in Asian countries. It remains unclear to what extent there are cultural differences in the interpretation of the EQ items, and therefore the difference between Asian and Western countries might partly stem from measurement invariance.

With regard to systemizing, we investigated the revised version of the SQ (SQ-R) in the present Dutch sample, and the obtained scores for both sexes were comparable to the other two studies using this version in a British sample (Baron Cohen et al. 2014; Wheelwright et al. 2006). In order to compare the SQ-R score to the scores of the other countries that made use of the original 40-item SQ, we recalculated the SQ-R score [SQ = (SQ-R/75) × 40]. The recalculated SQ-R scores of the present sample as well as the British samples were slightly higher compared to the scores of the other countries making use of the original 40-item SQ. This is most likely explained by the characteristics of the SQ-R, which includes more items that are less specific for males and more suitable for both sexes. We therefore recommend not to directly compare the SQ to the SQ-R. Reviewing the current literature on the SQ showed that Asian samples (for both student and community samples) score similar to Western samples on SQ and that the sex differences are also similar in magnitude, ranging from medium to large across international studies. Concerning cross-cultural stability of the SQ, it can be concluded that, different from EQ, SQ is stable regarding mean scores and sex differences across cultures.

In the present study good reliability and validity of especially the short 28-item version of the EQ was replicated (the Dutch EQ, scoring key, and norm table can be requested from the corresponding author). The 28-item EQ had overall good validity, as was evident by (a) significant sex differences with medium effect size, by (b) significant differences between males with and without an ASD diagnosis with mostly large effect sizes, by (c) weak to large positive correlations with a questionnaire assessing the enjoyment and importance of friendships and interest in other people (FQ), and by (d) negative correlations with the AQ in an ASD sample. A three-factor structure with the factors CE, EE and SS could be supported by factor analysis on 28 out of the 40 original items that had been proposed in previous psychometric studies on the EQ (Berthoz et al. 2008; Dimitrijevic et al. 2012; Lawrence et al. 2004; Muncer and Ling 2006; Preti et al. 2011). The 28-item EQ had overall good consistency and good test–retest reliability across a time span of 15 months. On a subscale level, SS had a lower consistency and lower intercorrelations compared to the CE and EE scales which could be due to the lower number of SS items (6) compared to the CE (11) and EE (11) scales. Another factor that may play a role in its low reliability is that the SS scale mainly consists of reversed items and that reversed items were shown to have overall lower consistency than forward items. The moderate intercorrelations with the SS scale could therefore be due to the lower reliability of this scale, but could alternatively suggest that the SS scale is related to, but nevertheless different from the general construct of empathy. Another point of discussion on the subscale level is that unlike the CE and SS subscale, the EE scale had only moderate test–retest reliability. We speculate that the EE score may in addition to empathic *trait* factors, also measure *state* factors. The majority of its items refer to feelings in relation to other people that may vary with the current social context or the affective state. Clinicians and researchers should therefore be cautious in interpreting the EE subscale as a fixed emotional empathic trait, and consider the social context or affective state at the time of the assessment.

The SQ-R also appeared to be a reliable and valid measure (the Dutch SQ-R, scoring key, and norm table can be requested from the corresponding author). The factor analysis on the 75-item SQ-R (Wheelwright et al. 2006) demonstrated that a one-factor structure was preferable, because no statistical or psychological meaningful clusters were found in a multifactor solution. Given the high internal consistency of the total scale, we decided in line with Wheelwright et al. (2006) that it was more appropriate to interpret SQ-R as a single scale without any specific subscales. The test–retest reliability of the SQ-R was also good, and divergent validity was reasonable as indicated by weak to moderate negative correlations with EQ and FQ in the community sample, and a weak positive correlation with AQ in the ASD sample. Although divergent validity of SQ-R appeared reasonable, convergent validity was not tested in this study which would be necessary for further validation of the SQ-R. Some studies with the original SQ did demonstrate good convergent validity, as higher SQ scores go along with higher scores on visuospatial tasks, such as mental rotation and ball targeting (Cook and Saucier 2010; Ling et al. 2009). With regard to criterion validity, typical sex differences of medium effect size could be demonstrated, but surprisingly no differences were found between males with and without ASD. Patients with ASD scored in the same range as the males of the norm group. Furthermore, ROC analyses exploring the accuracy of SQ-R in detecting males with ASD yielded poor predictive validity for the SQ-R.

In the light of the EMB hypothesis of ASD, the outcomes in this study provide support for reduced empathy in ASD but not for increased systemizing. The sole use of SQ-R scores was not predictive of having ASD or not. The EQ and ‘brain type’ were better predictive measures, as ROC analyses revealed that both EQ and D could detect patients with ASD above chance level. However, the combinations of sensitivity and specificity were suboptimal, so the instruments are not suited for predictive or diagnostic purposes. It must be noted that the predictive value of ‘brain type’ is most likely carried by the predictive value of EQ, which partly constitutes the ‘brain type’ measure. Furthermore, only a weak negative association between the EQ and SQ-R was found in the community sample and this correlation was absent in the ASD sample. This implies that there is only a weak trade-off between empathizing and systemizing, which is inconsistent with the EMB hypothesis stating that these cognitive styles are complementary. The latter findings could however relate to the inclusion of a relatively heterogeneous ASD sample (see “Limitations” section). Wheelwright et al. (2006), for example, did find a stronger negative association between EQ and SQ-R in a sample of ASD patients compared to a typical group, suggesting a stronger trade-off between empathizing and systemizing in patients with ASD. Other studies did provide support for increased systemizing in ASD (Baron Cohen et al. 2014; Wakabayashi et al. 2007; Wheelwright et al. 2006). More support for the systemizing part of the EMB theory in adult samples is necessary, not only by means of the SQ but also by neuropsychological assessments.

### Limitations

The actual sex differences for empathy and systemizing could be smaller than the sex differences reported in this study because of several reasons. Firstly, regarding empathy, participants may fill-out the EQ in a social desirable or sex-stereotypical way. Previous studies found somewhat smaller sex differences for EQ when controlling for social desirability (Berthoz et al. 2008; Preti et al. 2011) and an association was found between EQ and social desirability, which is larger in females than males (Vellante et al. 2013). We expect that the influence of social desirability is smaller in the short 28-item version of the EQ, because this version excludes those items with high loadings on social desirability (see Lawrence et al. 2004). Secondly, it is not known whether males and females differ in the way they interpret the items of the EQ and SQ-R (i.e. to what extent there is measurement invariance), and therefore part of the sex difference could be due to measurement artefacts. As these limitations specifically apply to self-report measures, it is advisable to rely not only on self-report measures for the assessment of empathy and systemizing, but to also include more objective measures, such as social-cognitive tasks (e.g. Vellante et al. 2013). Finally, the sample of the present study was not randomly selected from the community and may therefore suffer from a self-selection bias. It is possible that empathic males and females are more likely to participate in studies like these. However, since the mean scores and the magnitude of the sex differences are in line with other international studies, we do not consider this limitation as a serious threat to the validity of the findings.

No back-translation has been performed on the Dutch EQ and SQ-R translations, which may have caused minor differences between the Dutch versions and the original English versions. These minor differences are not likely to have influenced the validity of the questionnaire, because the psychometric properties of the Dutch questionnaires were very similar to those reported in previous studies.

The included high functioning ASD sample can be described as a heterogeneous sample including the different conditions from the broad autistic spectrum, ranging from mild to severe. Although the patients were all diagnosed with a DSM-IV classification in the autistic spectrum, a large proportion had not been assessed with an instrument that is regarded as gold standard for the assessment of ASD, such as the ADOS. The majority did not achieve the proposed AQ cut-off score of 32 by Baron-Cohen et al. (2001). Interestingly, the vast majority of the patients scoring below this cut-off were rated as having clinical problems in the autistic spectrum according to their friends, families or professionals on the ADOS or SRS-A. However, in the present study such other-report measures were unfortunately not available for all patients in order to objectify their autistic spectrum problems. The heterogeneity of the sample, however, might have influenced the results in that respect that even stronger EQ differences and actual SQ-R differences could be found in more severe ASD samples.

### Clinical Use

Although lowered EQ is a consistent finding in ASD, the EQ cannot be used to predict or diagnose whether a person has ASD, because its predictive value appeared insufficient for this purpose. Following the methodological framework for assessing health indices (Kirshner and Guyatt 1985), the EQ is not regarded a discriminative or predictive measure, but is rather useful as an evaluative measure. It yields information about an individual’s experience of empathy and the individual’s strengths and weaknesses regarding particular aspects of empathy. The EE subscale should be carefully interpreted in the light of the social context and affective state at the time of assessment, because its test–retest reliability appeared only moderate. Regarding the SQ-R, poor predictive validity was found in a heterogeneous sample of ASD patients. Based on the present study, we therefore recommend to interpret the SQ-R score always in relation to EQ, because SQ-R may lie in the normal range, whereas the discrepancy between empathizing and systemizing in the brain may be large. As for EQ, the SQ-R should merely be viewed as an evaluative measure of an individual’s systemizing style.

The EQ and SQ are self-report measures that depend on the participant’s capacity of self-reflection. Although healthy individuals may in general be well able to reflect upon their own cognitive style, i.e. possess the ability of meta-cognition, this ability may be limited in patients with autism. For example, patients with Asperger syndrome were shown to be impaired in self-reflection and self-awareness (Jackson et al. 2012). When using the EQ and SQ as assessment tools (as well as other self-report tools such as the AQ and SRS-A), they can therefore only be interpreted reliably when the examinee (e.g. a patient with ASD) disposes of good self-reflection abilities. In this context, it is important to consider that self-awareness is regarded as an important part of empathy, because it allows an empathic person to clearly differentiate between his/her own experience and that of the person being observed (Decety and Meyer 2008). This means that patients with ASD who are more impaired in self-reflection abilities may also suffer from greater impairments in empathy, while at the same time they might overestimate their empathic skills on self-report questionnaires like the EQ. Therefore it is important to consider self-reflection or meta-cognitive skills when assessing or interpreting self-reports of empathy. This issue also underscores the importance of using other informants for assessing empathy (Johnson et al. 2009).

## Conclusion

This study shows good reliability and validity of the Dutch 28-item EQ and 75-item SQ-R. These measures can therefore be used to reliably assess a person’s empathizing and systemizing cognitive style, although self-reflection skills should be taken into consideration when interpreting the scores. Regarding the EQ, a three-factor structure was replicated with the subscales Emotional Empathy, Cognitive Empathy and Social Skills. These subscales allow a more refined evaluation of a person’s empathic skills. The test–retest reliability of the Emotional Empathy scale was moderate, suggesting that it measures a mixture of state and trait emotional empathy. The EQ and SQ-R together, provide information about a person’s brain type (e.g. empathizing/female brain, systemizing/male brain, and balanced brain). Patients with ASD scored lower on EQ and ‘brain type’, which is in line with the EMB hypothesis of autism (Baron-Cohen 2009), however patients did not differ from the males of the norm group with regard to the SQ-R score. Reviewing the international literature on EQ and SQ revealed that (a) SQ appears to be stable in mean scores and sex differences across cultures, that (b) EQ is stable in Western countries as well, but that (c) EQ is characterised by a lower stability and sensitivity for sex differences in Asian countries.
